# Management of Unerupted Maxillary Deciduous Central Incisor: A Case Report

**DOI:** 10.5005/jp-journals-10005-1236

**Published:** 2014-04-26

**Authors:** Karam Abu Shakra

**Affiliations:** Consultant, Department of Pediatric Dentistry, King Hussein Medical Center, Royal Medical Services, Amman, Jordan

**Keywords:** Eruption, Primary incisor, Fibroma

## Abstract

Failure of eruption of primary teeth can be considered rare, especially in maxillary anterior teeth. The problem can be either mechanical obstruction of eruption or a failure of the eruption mechanism. This case report presents failure of eruption of the maxillary right deciduous central incisor in a 4-year-old girl. The unerupted primary tooth was removed surgically. The histological finding revealed fibroma with reactive giant cells. Periodic follow-up visits were advised to monitor the developing dentition and to ensure enough space for the permanent incisor.

**How to cite this article: **Shakra KA. Management of Unerupted Maxillary Deciduous Central Incisor: A Case Report. Int J Clin Pediatr Dent 2014;7(1):58-60.

## INTRODUCTION

Tooth eruption is a physiologic process by which a tooth moves from its site of development to its final functional position in the oral cavity.^1^ Eruption failure may occur in either the primary or the permanent dentition, it can affect one or a number of teeth, and can be partial or complete, depending upon the underlying etiology.^2^ Failure of eruption can also be primary failure which means that the tooth fails to erupt as a result of malfunction of the eruption mechanism, or it could be secondary, meaning that the tooth after eruption becomes ankylosed and loses its ability to maintain the continuous eruptive potential.^3^

Failure of eruption of primary teeth has been reported to be very rare. Prevalence of impacted primary teeth has been reported to occur in 1:10,000 times, most commonly involving the primary second molars and usually affecting females.^4^

Unerupted maxillary incisors are of concern to both the dentist and the parents since this can have a big Influence on dental and facial esthetics. In addition for its psychological effects, this can cause speech and mastication problems. Therefore, it is important to detect and manage the problem as early as possible.^5^

## CASE REPORT

A 4-year-old girl attended the Pediatric Dentistry Clinic at Queen Alia Military Hospital (Jordanian Royal Medical Services) complaining of unerupted upper right central incisor tooth. As reported by the parents, the tooth had never erupted, and they could not recall any trauma to the oral cavity or the head and neck region. The girl had no medical problem and had a normal past family history. The patient was not taking any medications. There was no history of caries or intraoral infection.

Extraoral examination revealed no abnormal signs, no asymmetry and no lymphadenopathy.

The intraoral examination showed primary dentition stage, where all the primary teeth were present except the maxillary right central incisor. There was a labial swelling (bulge) in the region of the unerupted tooth, which was asymptomatic. The contralateral maxillary left central incisor had erupted normally.

**Fig. 1:**
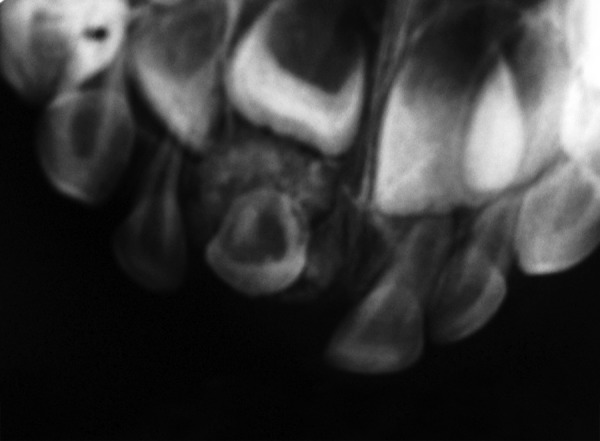
Preoperative X-ray showing the unerupted primary incisor and the surrounding bone

Anterior occlusal and periapical radiographs were taken. A single radiopaque structure representing the crown of the impacted tooth was present. This was surrounded by dense radiopaque irregular bone. Root formation was incomplete at that time. The successor tooth was present with wide radio-lucent pulp chamber. No odontomes and no supernumerary teeth were present (Fig. 1).

Considering the age of the child, the position of primary central incisor, and developmental stage of the permanent central incisors, it was decided to surgically remove the impacted primary central incisor. There was a little chance for normal spontaneous eruption of the tooth and also because it appeared that it may interfere with the development and eruption of the permanent tooth. Informed consent was taken from the child's parents before the surgery.

**Fig. 2:**
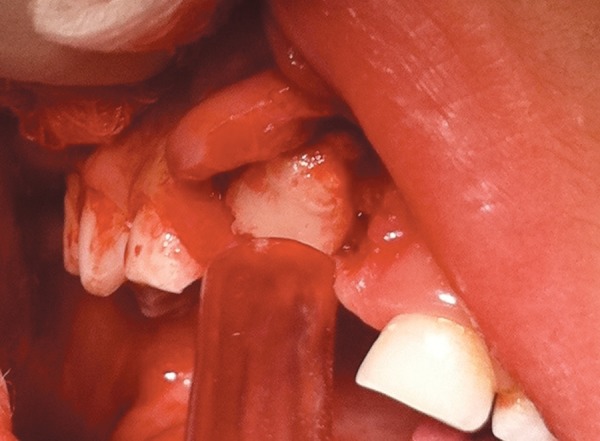
A small mucoperiosteal flap made by the periodontist

**Fig. 3:**
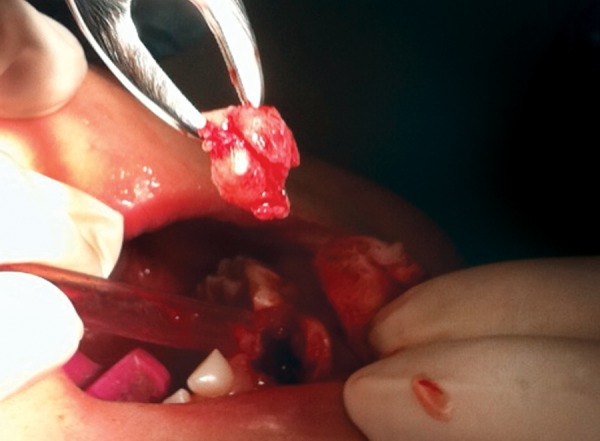
The unerupted deciduous incisor with the surrounding tissues

The patient was very cooperative, so the surgical pro­cedure was carried out under local anesthesia. A minimal approach was used in which a small semilunar full thick-ness flap was made by the periodontist, in order to preserve the interdental papillae (Fig. 2). The deciduous crown, surrounded by thin overlying bone, and soft tissue was removed (1 × 1 × 0.5 size) without disturbing the underlying successor (Fig. 3). However, there was no root of the deciduous central incisor. The socket was cleaned and curettaged. Suturing was done with resorbable stitches, and oral hygiene instructions were given to the parents. The primary tooth with the sur­rounding tissues was placed in formalin and then was sent to the histopathology laboratory. The microscopic report revealed presence of fibroma with reactive giant cells and no signs of malignancy.

**Fig. 4:**
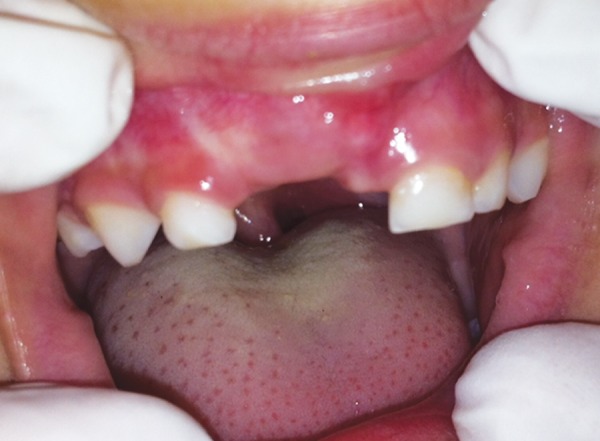
The labial bulge postoperatively

The patient was given regular postoperative follow-up visits. The healing was good and the labial bulge had reduced (Fig. 4). The patient had no complaints. An intraoral periapical radiograph 2 months was normal. There was also enough space to accommodate the permanent maxillary right central incisor so a partial denture was planned to be placed later for esthetic and psychological reasons. Further periodic recall visits were scheduled to ensure normal eruption of permanent incisor and to monitor the developing dentition.

## DISCUSSION

Failure of eruption can be considered rare in primary teeth; it is usually more common in permanent teeth and occurs because of many etiologic factors, such as mechanical obs­truction in the path of eruption, including odontomas, odon-togenic tumors, ankylosis, trauma and dentigerous cysts. Malpositioning of the tooth germ is another factor, either due to trauma or unknown reasons, which may cause impactions due to lack of space or primary failure of eruption.^6^

In this case report, a rare case of primary failure of eruption of primary central incisor localized to the maxilla was presented. Failure of eruption was associated with giant-cell fibroma, which is a benign non-neoplastic lesion, not associated with trauma or irritation. It is a localized reactive proliferation of fibrous connective tissue. It occurs in the first three decades of life and predominates in females. The most characteristic histological feature is the presence of large spindle-shaped and stellate-shaped mononuclear cells and multinucleated cells.^7^

There are few case reports in the literature regarding unerupted primary anterior teeth. Kapur A et al in 2008 reported an unusual case of inverted impacted primary incisors.^4^

The inverted primary maxillary central incisors were surgically extracted and prosthetic rehabilitation was done to improve the esthetics, speech and psychosocial behavior of the child. While Das UM et al in 2002 published a case of unerupted maxillary canine associated with compound composite odontoma.^8^ Surgical exposure was done and the mechanical obstruction was eliminated to allow spontaneous eruption of the permanent tooth. Lambert M and Rothman DL also reported an unusual impaction of primary lateral incisors.^9^

Bodner L and Horowitz I studied impaction of primary incisor.^10^

## CONCLUSION

It is important to carefully monitor the eruption of primary and permanent teeth for early identification and manage­ment of any abnormality in tooth eruption. Unerupted deciduous teeth with defects should be extracted at the proper time taking into consideration the development of the successor tooth and the space relationships in the permanent dentition.
